# Zika Virus IgM 25 Months after Symptom Onset, Miami-Dade County, Florida, USA

**DOI:** 10.3201/eid2512.191022

**Published:** 2019-12

**Authors:** Isabel Griffin, Stacey W. Martin, Marc Fischer, Trudy V. Chambers, Olga L. Kosoy, Cynthia Goldberg, Alyssa Falise, Vanessa Villamil, Olga Ponomareva, Leah D. Gillis, Carina Blackmore, Reynald Jean

**Affiliations:** Florida Department of Health in Miami-Dade County, Miami, Florida, USA (I. Griffin, C. Goldberg, A. Falise, V. Villamil, O. Ponomareva, R. Jean);; Centers for Disease Control and Prevention, Fort Collins, Colorado, USA (S.W. Martin, M. Fischer, T.V. Chambers, O.L. Kosoy);; Bureau of Public Health Laboratories, Miami (L.D. Gillis);; Florida Department of Health, Tallahassee, Florida, USA (C. Blackmore)

**Keywords:** Zika virus, immunoglobulin M, IgM, MAC-ELISA, IgM antibody capture ELISA, Miami-Dade County, Florida, United States, viruses, vector-borne infections, mosquitoborne viruses, antibody survival, flaviviruses, antibody persistence

## Abstract

We assessed IgM detection in Zika patients from the 2016 outbreak in Miami-Dade County, Florida, USA. Of those with positive or equivocal IgM after 12–19 months, 87% (26/30) had IgM 6 months later. In a survival analysis, ≈76% had IgM at 25 months. Zika virus IgM persists for years, complicating serologic diagnosis.

Diagnosis of Zika virus infection is accomplished by testing for viral RNA or IgM and neutralizing antibodies (*1*). A cohort study of 62 confirmed Zika virus cases from the 2016 outbreak in Miami-Dade County, Florida, USA, demonstrated that Zika virus IgM remains detectable in most (92%) persons 12‒19 months after symptom onset (*2*). We estimated the proportion of persons with detectable Zika virus IgM up to 25 months after initial illness onset.

## The Study

We included persons residing in Miami-Dade County who had confirmed Zika virus disease with symptom onset during June‒October 2016 and had participated in a previous prospective cohort study (*2*). Of the original 62 patients, we asked all 57 patients with positive or equivocal Zika virus IgM results at 12‒19 months after symptom onset to provide another specimen 6 months later. We obtained written consent for the additional specimen from study participants. We tested all serum specimens at the Centers for Disease Control and Prevention (Fort Collins, Colorado, USA) by the IgM capture ELISA for Zika virus (*3*–*5*).

We used SAS version 9.4 (https://www.sas.com) to manage and analyze the data and performed a nonparametric survival analysis (i.e., PROC ICLIFETEST) for interval-censored data to estimate the duration of Zika virus IgM detection. For this procedure, we considered survival to be the detection of Zika virus IgM (a positive or equivocal result). We included the IgM results of specimens from all 62 original participants collected 12‒19 months after symptom onset and the IgM results from all follow-up specimens acquired in the survival analysis. The Florida Health Institutional Review Board (Tallahassee, Florida, USA) approved this study.

Of 57 persons with positive or equivocal Zika virus IgM results at 12‒19 months after symptom onset, 30 (53%) provided a follow-up specimen. The median time of specimen collection after symptom onset was 21 (range 18–25) months; 5 (17%) patients provided a specimen at 18 months after symptom onset, 1 (3%) at 19 months, 6 (20%) at 20 months, 9 (30%) at 21 months, 3 (10%) at 22 months, 3 (10%) at 23 months, 1 (3%) at 24 months, and 2 (7%) at 25 months.

Demographics and clinical characteristics of the 62 participants in the original study were previously reported (*6*). Of the 30 who provided an additional follow-up specimen, the median age at symptom onset was 45 (range 22–70) years; all were adults >18 years of age. Fifteen (50%) were female, and 14 (47%) were Hispanic. After reviewing case investigations, we found that 13 (43%) of these participants reported no international travel (outside of the continental United States) during the 2 years before collection of the last specimen.

Of the 30 participants who provided a follow-up specimen, 19 (63%) were positive for Zika virus IgM, 7 (23%) had an equivocal result, and 4 (13%) were IgM seronegative. Compared with results from the specimen collection 6 months earlier, 20 (67%) remained positive for Zika virus IgM, 2 (7%) remained Zika virus IgM equivocal, 4 (13%) transitioned from Zika virus IgM positive to equivocal, and 4 (13%) transitioned from Zika virus IgM equivocal to negative; no participants switched from Zika virus IgM positive to negative. Because of the small sample size, we were unable to assess whether age group, race, or ethnicity was associated with Zika virus IgM results. When we used all available test results from the 62 participants, a survival analysis indicated that 93% (95% CI 82%‒97%) of participants had detectable (positive or equivocal) Zika virus IgM at 14 months after symptom onset, 91% (95% CI 81%‒96%) at 17 months, 81% (95% CI 69%‒89%) at 22 months, and 76% (95% CI 57%‒88%) at 25 months (Figure).

**Figure:**
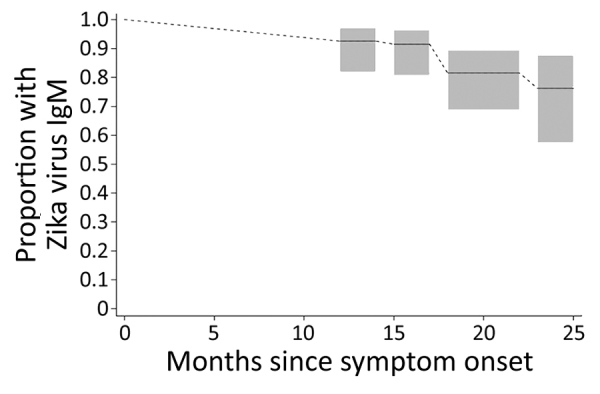
Estimated proportion of persons with detectable Zika virus IgM up to 25 months after symptom onset among persons with PCR-confirmed Zika virus disease, Miami-Dade County, Florida, USA. Detectable Zika virus IgM was defined as a positive or equivocal result on IgM capture ELISA. Interval-censored nonparametric survival analysis probability estimates and 95% CIs (gray boxes) are shown.

## Conclusions

Our findings suggest that approximately three quarters of persons with PCR-confirmed symptomatic Zika disease still have detectable IgM at 25 months after initial illness onset. The prolonged detection of IgM after Zika virus infection is consistent with previous findings for related flaviviruses (*6*–*10*). Our findings are specific to the Centers for Disease Control and Prevention IgM capture ELISA for Zika virus, which targets the premembrane and envelope glycoproteins; other available IgM serologic assays targeting other Zika virus proteins might not produce comparable findings (*3*). In addition, these results are only representative of symptomatic Zika cases; whether persons with asymptomatic Zika virus infections exhibit similar Zika virus IgM persistence is unknown. IgM persistence needs to be assessed with other serologic assays for both symptomatic and asymptomatic Zika virus cases to determine the full duration of Zika virus IgM after infection.
